# Assessment of adherence behaviors for the self-reporting of occupational exposure to blood and body fluids among registered nurses: A cross-sectional study

**DOI:** 10.1371/journal.pone.0202069

**Published:** 2018-09-26

**Authors:** Yifang Yi, Sue Yuan, Yinglan Li, Dan Mo, Li Zeng

**Affiliations:** 1 Division of Otorhinolaryngology, Xiangya Hospital of Central South University, Changsha City, Hu’nan Province, China; 2 Xianga Nursing School of Central South University, Changsha City, Hu’nan Province, China; 3 Department of Infectious Disease, Xianga Hospital of Central South University, Changsha City, Hu’nan Province, China; 4 Department of Nursing Management, Xiangya Hospital of Central South University, Changsha City, Hu’nan Province, China; 5 Department of Burns Surgery, Xiangya Hospital of Central South University, Changsha City, Hu’nan Province, China; Public Library of Science, UNITED KINGDOM

## Abstract

**Background:**

In China, register nurses (RNs) have a high risk of occupational exposure to blood/body fluids. The adherence behavior related to self-reporting of occupational exposure needs to be evaluated to protect RNs from healthcare-related infections.

**Objectives:**

To assess adherence behaviors for self-reporting of occupational exposure to blood and body fluids among RNs and identify factors affecting self-reporting in Hunan Province, China for developing upgraded strategies.

**Methods:**

Study participants, randomly selected from six tertiary hospitals in Changsha City, completed a structured questionnaire. Frequencies and percentages were used to describe basic demographic data. One-way analysis of variance was performed to assess whether adherence behaviors were correlated to each other; the multivariate logistic regression analysis was performed to identify factors associated with reporting exposure to blood/body fluids.

**Results:**

In total, 548 RNs completed the questionnaire. All participants experienced sharp object injuries at least once during their career; 65.88% of participants were exposed to blood/body fluids thrice, and 31.2% experienced 1–5 occupational exposures in the past month. However, only 14.6% of participants submitted a blood/body fluid exposure report to a supervisor/official after every incident. Blood/body fluid exposure was associated with the non-usage of safety protocols. Only 10.2% of participants believed the employer paid more attention to needle-stick injuries (P<0.01) than to other injuries. Most participants (73.5%) reported the absence of psychological support after injuries (P<0.01). Nine personal and management factors were observed to be closely related to underreporting behavior.

**Conclusion:**

The prevalence of exposure to blood/body fluids among RNs was high, and the underreporting rate was likely substantially underestimated. Safety-engineered devices must be adopted to decrease the prevalence of sharp object injuries. To encourage employees to report occupational exposure events, a series of hospital-wide actions need to be adopted.

## Introduction

Nurses have a high risk of occupational exposure, which is associated with the transmission of >20 pathogens [1–5], including hepatitis B virus (HBV), hepatitis C virus (HCV), and human immunodeficiency virus (HIV). In China, HBV and HCV infections are considered epidemic. Data based on China’s sixth nationwide census revealed that approximately 100,000,000 Chinese citizens are chronic HBV carriers or patients, and 30,000,000 citizens have HCV infection [6,7]. The HIV infection prevalence is also concerning. At the end of 2013, in China, of 263,000 people living with HIV, 174,000 developed acquired immune deficiency syndrome, and 136,000 deaths were related to HIV [8,9,10]. Our recent results of monitoring occupational exposure among healthcare workers (HCWs) over a two-year monitoring period [11,12] showed that 47.65% of HCWs with exposure to blood/other body fluids had detectable levels of bloodborne pathogens, including HBV, HCV, or HIV. In addition, 25.6% of source patients were positive for hepatitis B virus surface antigen (HBsAg), 8.7% for HCV RNA, and 3.5% for HIV. The proportion of source individuals with bloodborne pathogens (47.65%) was far higher than the “one in five” previously reported [13]. Finally, in our study, two individuals exposed to HCV were seropositive for HBV one month after the incident and recovered six months after combination antiviral therapy with pegylated interferon and ribavirin.

Following percutaneous injury, 6–30 of 100 HCWs, 10 of 100 people, and 0–1 of 100 people are at a risk of acquiring HBV infection [14], HCV infection [15,16], and HIV infection [17,18], respectively. Therefore, HBV or HIV exposure does not necessarily result in infections. A very short time period, called a “window of opportunity,” may exist for preventing seroconversion after exposure. A case-control study in 1997 revealed that seroconversion rates among HCWs receiving zidovudine prophylactically after exposure to HIV were reduced by approximately 81% [19]. If the source patient is HBsAg positive, the odds of acquiring HBV infection among exposed workers who are not vaccinated or have antibody levels <10 mIU/ml but have been administered hepatitis B immunoglobulins (0.6 ml/kg, preferably within 24 h) and a vaccine at a different site can be reduced by 90% [20]. Regarding post-exposure prophylaxis (PEP) for HCV, an early antiviral therapy during the acute phase of infection is associated with >95% of resolved infections [21,22].

Thus, timely PEP use against infectious agents effectively reduces the likelihood of seroconversion after events with a high risk of exposure to HBV or HIV. This is based on timely incident reporting, which is the only way to ensure that exposed individuals receive a PEP regimen and counseling. Otherwise, an exposed HCW could miss the opportunity to avoid occupational infection development.

However, the underreporting of exposure to blood/body fluids was common. A large number of studies showed that ≥80.6% of HCWs experienced percutaneous exposure, but only ≤7.8% reported their injuries [23–26]. Meanwhile, only 10.2% of healthcare settings have developed and implemented a hazard reporting protocol that describes in detail where and how HCWs should seek treatment after occupational exposure to ensure timely medical treatment [23].

Currently, the self-reporting compliance among RNs in this study region remains unknown. Thus, this study aimed to assess the adherence behavior for self-reporting of occupational exposure to blood and other body fluids among RNs and identify factors that contribute to self-reporting following exposure to upgrade strategies to effectively prevent healthcare-related infections in this population.

## Materials and methods

### Settings, study design, and participants

We used a two-stage stratified proportional random sampling procedure to obtain a regional representative sample. According to the government urbanization index, 11 tertiary general hospitals in Changsha City were divided into four municipal, four provincial, and three ministerial institutions, employing a total of 8,483 RNs. Our research areas included two municipal, two provincial, and two ministerial institutions, which were randomly selected from the master samples at 50% proportion.

The study population was limited to full-time RNs working in these tertiary hospitals; nurses who did not provide patient care (e.g., the director of the nursing department and trainees) were excluded. According to the 10% proportion, 586 RNs were selected from the six hospitals using a random number generated by a random number generator. These RNs were contacted through the directors of the nursing departments or nurse managers in charge of their departments. Data were collected from June 1 to August 31, 2015 and were analyzed anonymously. The above-stated settings predominantly treat critical patients, and there is an imbalance between demand and supply for nurses in these settings [24]. The prevalence rates of bloodborne pathogens such as HBV, HCV, and HIV are similar to the mean values in China. In addition, actual data on blood or body fluid exposure among RNs in these institutions are limited.

### Questionnaire and data collection

Based on the “HBV occupational preventive knowledge and behaviors survey form,” which was revised by Wang Hong-Hong, the survey questionnaire was compiled and revised after being pilot-tested in clinical settings and interviews with respondents. Ten experts evaluated the content and applicability of the revised version, and the Cronbach’s ɑ coefficient of internal consistency and retest reliability were 0.806 and 0.708, respectively.

The final questionnaire with 34 closed-ended questions and one open-ended question; it comprised sociodemographic characteristics and questions to enable the assessment of the knowledge on and practice of occupational exposure to blood and body fluids and management of exposure incidents. It particularly asked respondents to list the obstacles to reporting risk incidents and the number of exposure incidents in the last month. In this study, the age of the participants was classified into four categories: <25, 25–29, 30–34, and >34 years. We distributed questionnaires to all 586 participants, who were asked to complete them after signing a written informed consent form. To increase the response rate, an honorarium of nearly $2 USD was attached to the questionnaire. After one week, we called the selected facilities to ask the RNs to return the completed questionnaires.

### Statistical analysis

All statistical analyses were performed using SPSS PASW Statistics, version 18. Data are summarized as frequencies and percentages where applicable. We used one-way analysis of variance to assess whether adherence behaviors were correlated to each other. The odds ratio and 95% confidence interval were estimated from the multivariate logistic regression analysis to determine the association between influencing factors and reporting behaviors. We further performed multivariate logistic regression analyses using reporting behaviors as the dependent variable and personal and management factors closely related to underreporting behavior as independent variables. To reduce confounding bias, we adopted a backward linear regression analysis (α entry = 0.10, α removal = 0.15). A P-value <0.05 was considered statistically significant.

### Ethical considerations

The Ethics Committee of Central South University Hospital approved the study before initiating participant recruitment. Participants were informed about the study (its importance, risks, and prevention strategies) and were assured about the confidentiality of information. Written informed consent was obtained from each nurse using a form provided with the questionnaire.

## Results

### Sociodemographic characteristics (Table 1)

**Table 1:** Demographic and practice characteristics (n = 548).

Characteristic	N0. (%)
Gender	
Female	536 (97.8)
Male	12 (2.2)
Age (years)	
<25	249 (45.4)
25–29	195 (35.6)
30–34	62 (11.3)
> = 35	42 (7.7)
Tertiary general hospital	
Ministerial	286 (48.8)
Provincial	181 (30.9)
Municipal	119 (20.3)
Clinical department	
Emergency room	153 (27.9)
Emergency pediatrics room	59 (10.8)
Intensive care unit	145 (26.5)
Operating room	46 (8.4)
General medical ward	103 (18.8)
General surgical ward	42 (7.7)
Professional technical title	
Primary	317 (57.8)
Middle	160 (29.2)
Advanced	71 (13.0)
Work experience (years)	
<5	318 (58.0)
5–9	120 (21.9)
10–14	60 (10.9)
> = 15	50 (9.1)
Educational background	
Technical secondary degree	14 (2.6)
Junior college degree	302 (55.1)
Undergraduate or graduate degree	232 (42.3)
Anti-HBs	
Positive	300 (54.7)
Negative	248 (45.3)

In all, 548 of the 586 RNs returned the survey questionnaires with no missing data, for an overall response rate of 93.5%. An overview of the participants is provided in Table 1. All participants worked full time and provided day-to-day patient care in the clinical departments of tertiary general hospitals; 48.8% of them worked in ministerial general hospitals and 57.8% possessed a primary professional technical title. Furthermore, the majority (97.8%) of the participants were female. The average age was 27 years, with 81% of participants aged below 30 years.

### Self-reporting of occupational exposure to blood and body fluids (Table 2)

**Table 2:** Risks, exposures and self-reporting of blood/body fluid exposure.

Questions	Responses	N(%)
In your career, have you ever been exposed to blood or body fluids?	Yes	361(65.9)
	No	187(34.1)
In your career, have you ever been injured by a sharp object, such as a needle or scalpel?	Yes	548(100)
	No	0(0)
In the past one month, how many times have you been exposed to blood or body fluids?	0	377(68.8)
	1	120(21.9)
	2	35(6.4)
	3	8(1.4)
	4	3(0.6)
	5	5(0.9)
For how many of these exposures did you submit a blood/body fluid exposure reports?	all of these	80(14.6)
	most of these	101(18.4)
	a few of these	178(32.5)
	no one of these	189(34.5)

All respondents experienced at least one sharp object injury, such as with a needle or scalpel, during their healthcare career, and approximately two-thirds (65.9%) of the participants were exposed to blood/other body fluids. A large proportion (83.2%) of participants were aware of the need to complete accident reporting, while a minority (14.6%) of them submitted the blood/body fluid exposure reports to their supervisors or healthcare officials every single time. In all, over one-thirds (34.5%) of the RNs never completed an exposure report, although they were at a high risk for exposure to bloodborne pathogens.

### Factors associated with compliance to reporting behavior (Tables 3, 4 and 5)

**Table 3:** One-way analysis of demographic factors associated with the reporting behavior of blood/body fluid exposure.

Variable	Reporting all exposure (%)	Missing reporting exposure partial or full (%)	*χ*^2^	*P*
Age(years)				
<25	32(12.9)	217(87.1)	1.89	0.60
25–29	29(14.9)	166(85.1)		
30–34	12(19.4)	50(80.6)		
> = 35	7(16.7)	35(83.3)		
Professional technical title				
Primary	43(13.6)	274(86.4)	1.771	0.41
Middle	23(14.4)	137(85.6)		
Advanced	14(19.7)	57(80.3)		
Work experience (years)				
<5	46(14.5)	272(85.5)	1.098	0.78
5–9	15(12.5)	105(87.5)		
10–14	10(16.7)	50(83.3)		
> = 15	9(18.0)	41(82.0)		
Anti-HBs				
positivity	36(14.5)	212(85.5)	0.002	0.96
negativity	44(14.7)	256(85.3)		
Educational background				
technical secondary	2(14.3)	12(85.7)	Monte Carlo	0.57
junior	40(13.2)	262(86.8)		
undergraduate or graduate	38(16.4)	194(83.6)		

**Table 4:** One-way analysis of personal and management factors associated with the reporting behavior of blood/body fluid exposure.

Items	Reporting all exposure (n)		Missing reporting exposure partial or full (n)		*χ*^2^	*P*
	% of "yes" or "agree" responses	% of "unclear" or "no" responses	% of "yes" or "agree" responses	% of "unclear" or "no" responses		
Have experienced exposure to blood/body fluids at least once	13.9%(50)	16%(30)	86.1%(311)	84%(157)	7.96	0.02
Whether or not safety-engineered devices cause stick injuries	22.4%(34)	11.6%(46)	77.6%(118)	88.4%(350)	10.186	<0.01
The organization has provided training concerning occupational exposure	17.6%(66)	8.1%(14)	82.4%(309)	91.9%(159)	8.583	<0.01
Know PEP	19.4%(70)	5.3%(10)	80.6%(290)	94.7%(178)	19.765	<0.01
The organization has acted to prevent employees from exposure to blood/body fluids	18.9%(71)	5.2%(9)	81.1%(304)	94.8%(164)	20.678	<0.01
There are some policies about occupational exposure prevention	17.9%(75)	3.9%(5)	82.1%(345)	96.1%(123)	Monte Carlo *P* <0.01	
Know post-exposure reporting	17.3%(79)	1.1%(1)	82.7%(377)	98.9%(91)	16.19	<0.01
Reasons for not reporting: personal responsibility and bad luck	3.7%(3)	16.5%(77)	96.3%(79)	83.5%(389)	9.257	<0.01
Reasons for not reporting: did not know the reporting procedure	2.9%(3)	17.3%(77)	97.1%(101)	82.7%(367)	14.127	<0.01
Reasons for not reporting: the reporting procedure was cumbersome	9.9%(16)	16.5%(64)	90.1%(145)	83.5%(323)	3.972	0.05
Knows or does not know the reporting procedure	24.2%(66)	5.1%(14)	75.8%(207)	94.9%(261)	40.188	<0.01
One department responsible for supervising employees to ensure timely reporting	24.7%(63)	5.8%(17)	75.3%(192)	94.2%(276)	46.039	<0.01
One department responsible for the management of occupational exposure	21.6%(72)	3.7%(8)	78.4%(261)	96.3%(207)	Monte Carlo *P* <0.01	
The hospital has paid necessary attention to the exposure	50%(28)	10.6%(52)	50%(28)	89.4%(440)	67.176	<0.01
There is a manager responsible for meticulous follow-up	26.5%(39)	10.2%(41)	73.5%(108)	89.8%(360)	35.217	<0.01
There is a department responsible for psychological support	27.1%(42)	9.7%(38)	72.9%(113)	90.3%(355)	36.033	<0.01

**Table 5:** Multivariate logistic regression analysis of the factors impacting the reporting behavior of exposure to blood/body fluids (n = 548).

Variable	Number of respondents(n)		*P* value	OR	95% CI for exp(B)	
	% of "yes" or "agree" responses	% of "unclear" or "no" responses			Lower	Upper
Constant						
Whether or not safety-engineered devices cause stick injuries	27.74%(152)	72.26% (396)	0.006	2.156	1.241	3.747
Know PEP	65.69%(360)	34.31% (188)	0.056	0.533	0.279	1.017
The organization has acted to prevent employees from needle stick injuries	68.43%(375)	31.57% (173)	0.115	1.591	0.893	2.835
Know post-exposure reporting	83.21%(456)	16.79% (92)	0.086	6.05	0.777	47.093
Reasons for not reporting: personal responsibility and bad luck	14.96%(82)	85.04% (466)	0.029	0.245	0.069	0.865
Reasons for not reporting: the reporting procedure was cumbersome	29.38%(161)	70.62% (387)	0.149	0.62	0.323	1.187
Knows or does not know the reporting procedure	49.82%(273)	50.18% (275)	0.056	0.534	0.281	1.015
There is a department responsible for the management of occupational exposure	60.77%(333)	39.23% (215)	0.135	0.5	0.201	1.242
The hospital has paid necessary attention to the exposure	10.22(56)	89.78% (492)	0.001	0.442	0.271	0.721

According to the one-way analysis, the compliance to reporting behavior was not correlated to the demographic characteristics of the RNs, including their age, professional technical title, work experiences, level of anti-HBs, and educational background. The results are shown in Table 3. Seventeen personal or management factors were closely related to the adherence to underreporting behavior (P <0.05), and 16 of these are shown in Table 4. The final item was the question "Who will bear the burden of the cost associated with the occupational exposure?" Only one participant (3%) believed the injured may bear the burden of the cost associated with the occupational exposure reported, while an overwhelming majority (94%) of RNs stated that the financial burden of sharp object injuries should not be placed on the injured (Monte Carlo P<0.01). With respect to PEP regimens, 65.7% of the nurses were cognizant and quite a small proportion (19.4%) of them adhered to reporting procedures. This survey also showed that 21.6% of the participants did not know where to report their exposure and how to complete the form in a step-by-step manner (Table 4). Furthermore, the majority (68.4%) of RNs received training notices, but only 4.01% attended every training session.

Table 4 shows that nine personal and management factors in the model were closely related to underreporting behavior. These items are X2 (“Were the needles causing stick injuries safety-engineered?”), X4 (“Do you know the prophylaxis procedure to be followed after occupational exposure?”), X5 (“Has the organization taken action to prevent exposure to blood/body fluids among employees?”), X7 (“Do you know that you should submit a report after exposure to blood or body fluids?”), X8 (Reasons for not reporting: “I thought I was responsible for the exposure and I had bad luck”), X10 (Reasons for not reporting: “I thought I must have blood taken”, or “The reporting procedure was cumbersome”), X11 (“Do you know the reporting procedure?”), X13 (“Is there a department in the organization that is responsible for the management of occupational exposure?”) and X14 (“Do you think the hospital pays enough attention to blood/body fluid exposure cases?”) (*P* <0.01). Of these items, X2 and X7 were independent risk factors.

## Discussion

Few studies have discussed the critical aspect of the underreporting of occupational exposure to blood/body fluids. Therefore, we designed a cross-sectional study to investigate the same. Our results indicated a high prevalence of exposure to blood/body fluids among RNs in the study area, although compliance rates to reporting were low.

### Current status of occupational exposure to blood and body fluids

RNs were at a high risk of occupational exposure to blood and body fluids. All respondents reported that they experienced one or more sharp object injuries during their healthcare career, and that sharp object injuries were the primary cause of exposure to blood/body fluids. The incidence rate of sharp object injuries detected in the present study was higher than that reported among nurses in the United States (US) [27]. This result demonstrated that clinical nurses who provide day-to-day patient care are at a high risk of sharp object injuries and are particularly likely to contract bloodborne pathogen infections.

### HBsAb levels of the RNs

This study also showed that participants with lower incomes ensured their serum HBsAb levels were positive; this awareness rate was lower than that observed in Poland [28]. It is well-known that HBV vaccination is the fundamental manner in which HCWs can be protected against HBV infection [29], but approximately half of the staff members in the present study had low awareness on the importance of maintaining specific HBsAb levels. We also learned that these healthcare settings had no mandatory policies for HCWs to receive free HBV vaccination before joining the hospital.

### Factors associated with occupational exposure to blood and body fluids

The essential factor associated with needle-stick injuries was the use of unsafe safety-engineered devices. Safety-engineered needles causing stick injuries were ultimately entered into the model. This was sufficient proof that the implementation of safety-engineered devices was unsatisfactory in these centers despite the fact that the Guidelines for Prevention and Control for Occupational Exposure to Bloodborne Pathogens was issued more than seven years ago [30]. Data from the National Surveillance System for Health Care Workers in the US showed that hollow-bore needles and solid sharp objects are responsible for 94% of all sharp object injuries [31]. In China, recent evidence has shown that two-thirds of needle-stick injuries are caused by hollow-bore needles [32,33]. Thus, the use of safety-engineered devices is commonly considered among the most important strategies in needle-stick injury prevention and has been signed into the law in Europe and the US [34]. However, in China, this policy is only included in a recommended file named “Nursing practice standards for intravenous therapy.” Therefore, future research may reveal ways in which the legislation of safety-engineered device implementation in health services can be improved, so as to reduce the use of unsafe engineered devices, such as winged-steel needles, and efficiently reduce the incidence rates of needle-stick injuries and exposure to blood/body fluids.

### Adherence to self-reporting of exposure incidents

Our results showed that episodes of exposure to blood/body fluids were common among RNs. Similarly, several studies demonstrated that the report compliance for stick injuries among HCWs was relatively low [35–37], and one-third of needle-stick injuries were unreported [35]. While the rate of exposure reporting (always) in this study was very low, most RNs were aware of the importance of the same and also knew the pre-exposure prophylaxis, PEP, and reporting procedures. These hospitals also provided training to their employees and took action to prevent exposure to blood/body fluids among them. In such settings, RNs were still reluctant to report exposure incidents to the management as they thought the employer did not pay enough attention to needle-stick injuries. Logistic regression analysis identified this view from the item: “Do you think the hospital pays necessary attention to needle-stick injuries?” In this study, participants stated that they did not think there was a manager providing meticulous follow-up to the exposed individual, supervisor to ensure employees reported on time, and psychological support for exposed employees. There was no significant correlation between underreporting and age, profession, work experience, and educational background. In this study, the presence of a cumbersome reporting procedure was not the main cause for underreporting.

### Strategies to reduce the likelihood of underreporting for exposure to blood and body fluids

Underreporting may also reflect a relatively low utilization of PEP regimens. In general, HIV PEP initiation should start as soon as possible to be effective during a particular limited post-exposure time window [38,39]. Therefore, the key to reducing infection rates through occupational exposure to bloodborne pathogens among HCWs is to encourage them to report occupational exposure incidents through their own initiative.

All the above-stated factors related to underreporting indicated that employers should focus on designing a sharp object injury prevention program based on their physical workplace, which may have a direct impact on outcomes. This program should consist of a range of hospital-wide procedures to prevent exposure incidents and implement PEP as soon as possible. To achieve this goal, our study confirmed that the top priority should be to create a dedicated team responsible for this task. This team should comprise occupational safety and health experts who should provide follow-up to injured HCWs and ensure the implementation of PEP, and psychology experts to provide psychological support to employees, especially immediately after exposure. To ensure that every employee has access to the service easily, the telephone number of a 24-h online service should be posted in the workplace and on the local area network. Second, the evaluation of training effectiveness should be a topic of concern. A small proportion of participants were likely to find excuses to not participate in occupational health training programs. Although almost all participants in this study were aware that occupational exposure is a type of work-related injury, the majority of them remained reluctant to report this sort of occupational injury.

Therefore, diversifying the content of training programs, such as through including case studies of exposure and high-risk operations, to attract participants’ attention and engage their interest, may be an effective strategy. HCWs should attend several lectures by infectious disease experts focusing on the importance of producing and maintaining certain HBsAb levels, to prevent HBV occupational infection development. It is also important to highlight the need for PEP because low adherence to it may increase the risk of acquiring a bloodborne pathogen infection and threaten occupational safety [30]. In conclusion, it is important to provide regular diversified educational sessions to help students achieve a high level of self-awareness on the risk of exposure to blood/body fluids, and encourage them to practice safe working behaviors and report every exposure incident. Third, the costs of needle-stick injuries should include the direct costs related to the initial and follow-up treatment of exposed HCWs, which are estimated to range from $71 to almost $5,000, depending on the treatment provided [40–42]. Our survey revealed that this financial burden should not be out-of-pocket payments, as this plays a crucial role in the reporting of exposure events. Creating a culture of safety in the workplace and a blame-free environment for reporting is also very important [43]. A dedicated team should organize environmental risk assessments in the workplace, quarterly or more frequently, to identify risk factors and reduce occupational exposure, and also to improve awareness on preventing exposure to blood/body fluids among employees.

### Strengths and limitations

Our study had several limitations. First, this was a cross-sectional retrospective study, and the information regarding occupational exposure frequency was subject to recall bias. In addition, medical records or original reporting sources were not reviewed. Furthermore, discussions with administrators in charge of occupational exposure were not conducted, although management factors contributed to underreporting. Finally, this study was conducted at tertiary hospitals in Changsha City, and thus, the findings may not be generalizable to other medical institutions in China. Thus, intervention research, structure interviews, and surveys in other demographic groups are needed in the future.

In China, RNs in clinical units are predominantly young women. A national study published by The Ministry of Health of the People’s Republic of China indicated that at the end of 2016, there were 3.5 million RNs nationwide, and half of them had work experience of a maximum of five years. Men comprised only 1.8% this national population. Our study had similar findings, which may benefit other critical care hospitals, but not non-critical care hospitals.

## Conclusion

Overall, the prevalence of blood/body fluid exposure among RNs was very high. Owing to low self-protection awareness rates and limited incident reporting management, it is likely that the underreporting rate for exposure to blood/body fluids in this study may have been substantially underestimated. Therefore, we recommend the widespread adoption of safety-engineered devices to decrease the incidence of sharp object injuries in healthcare settings. The keys to protecting RNs against blood/body fluid exposure and promoting exposure incident reporting are to formulate a comprehensive program and create a dedicated team responsible for improving training effectiveness, as well as develop and maintain a culture of safety together with other preventive measures.
